# Expression and Y435-phosphorylation of Abelson interactor 1 (Abi1) promotes tumour cell adhesion, extracellular matrix degradation and invasion by colorectal carcinoma cells

**DOI:** 10.1186/1476-4598-13-145

**Published:** 2014-06-09

**Authors:** Konrad Steinestel, Silke Brüderlein, Jochen K Lennerz, Julie Steinestel, Klaus Kraft, Christian Pröpper, Viktor Meineke, Peter Möller

**Affiliations:** 1 Bundeswehr Institute of Radiobiology, Neuherbergstr. 11, 80937 Munich, Germany; 2 Institute of Pathology, University Hospital, Ulm, Germany; 3 Clinic of Urology, University Hospital, Ulm, Germany; 4 Institute of Pathology and Molecular Pathology, Bundeswehrkrankenhaus, Ulm, Germany; 5 Institute of Anatomy and Cell Biology, University of Ulm, Ulm, Germany

**Keywords:** Abelson interactor 1 (Abi1), Colorectal carcinoma, Extracellular matrix (ECM), Invasion, Metastasis

## Abstract

**Background:**

The Abelson tyrosine kinase (c-Abl) inhibitor STI571 (Glivec®) has been shown to effectively inhibit colorectal cancer cell migration and invasion. The c-Abl substrate abelson interactor 1 (Abi1) is a key regulator of actin reorganization and upregulated in colorectal carcinoma. The specific role of Abi1 in relation to extracellular matrix degradation and effects of targeting Abi1 phosphorylation have not yet been examined. Here, we investigated the role of Abi1 in relation to invasive properties in colorectal cancer.

**Methods and results:**

In 56 primary human colorectal carcinoma samples, we found overexpression of Abi1 in 39% at the invasive edge of the tumour, associated with an infiltrative phenotype and high-grade tumour cell budding (p = 0.001). To explore the role of Abi1 in vitro, we employed the Abi1 expressing and *KRAS*-mutated CHD1 model and performed matrix degradation assays that showed Abi1 localization at specific sites of matrix degradation. Moreover, quantification of matrix dissolution demonstrated suppression after RNAi knockdown of Abi1 by 95% (p = 0.001). Importantly, treatment with STI571 did abolish Abi1 Y435-phosphorylation, suppressed the matrix dissolution, decreased fibronectin attachment, and suppressed cell invasion through reconstituted extracellular matrix.

**Conclusion:**

Our data indicate that phosphorylated Abi1 contributes to the invasive properties of colorectal cancer.

## Background

Colorectal carcinoma (CRC) is the second leading cause of death from cancer in the western world in both women and men [1]. Patient prognosis is in most cases determined by the ability of tumour cells to invade into surrounding tissue, gain access to blood vessels and form distant metastases after dissemination with the blood stream. Accordingly, a dissociative tumour growth pattern with single-cell infiltration and high-grade tumour cell budding has been shown to be associated with poor prognosis in colorectal cancer [2,3].

Tumour cells facilitate extracellular matrix (ECM) degradation and invasion through the formation of specialized cytoplasmic protrusions, so-called invadopodia [4]. These consist of parallel actin bundles on the basis of a branched actin meshwork and are enriched in proteins that are involved in actin remodelling, including Cortactin, formins of the Diaphanous family and class I nucleation-promoting factors (NPFs) such as WASP or N-WASP [5]. Actin reorganization is tightly regulated; actin-related proteins (such as Arp2/3) that facilitate actin branching underlie the control of signalling cascades modulating the activity of NPFs and small GTPases [6]. Besides providing the structural basis for invadopodia, branched actin filaments give rise to broad-based lamellipodia that are a prerequisite for cellular migration [5,6].

Abelson interactor 1 (Abi1) is a 65kD-substrate of the eponymous Abelson non-receptor tyrosine kinase (c-Abl). The protein is almost ubiquitously expressed with a main focus in the central nervous system [7]. It has been shown that Abi1 plays a central role in synaptic maturation, since knockdown experiments in cultured rat hippocampal neurons led to an immature, filopodia-like synaptic phenotype [8]. In a previous study, we could show that heterogeneous nuclear ribonucleoprotein K, a 65kD protein that has the ability to act as a translational regulator via binding to mRNA transcripts, interacts with Abi1 in hippocampal neurons and exerts a similar effect on synaptic maturation [9]. The role of Abi1 in cytoskeletal dynamics during lamellipodia formation has been linked to its ability to join the WAVE multiprotein complex including Abi1, Nap1, HSPC300 and Sra/PIR121 [10,11]; the active complex brings bound Arp2/3 and actin monomers into close proximity, thus enhancing actin polymerization [12]. Accordingly, Abi1-deficient mouse embryos show malformations in the developing heart and brain and die around embryonic day 11; furthermore, they show decreased integrity and function of the WAVE complex [13]. Interestingly, Abi1 has also been shown to interact with the PI3-Kinase, enhancing cytoskeletal reorganization processes in support of cellular migration via activation of Rac activity [14,15]. In invading breast cancer cells, Abi1 is required for invadopodia formation and matrix-metalloproteinase 9 secretion [16]. Some effects of Abi1 on cytoskeletal reorganization are dependent on phosphorylation of the protein, and interestingly, application of the Abelson tyrosine kinase inhibitor STI571 (Glivec®) has been previously found to effectively suppress colorectal cancer cell invasion as well as metalloproteinase-dependent matrix degradation by breast cancer cells [17-19]. Significant antiinvasive effects of STI571 on colon cancer cells have been observed upon application of subtoxic concentrations of the drug in several reports, while the underlying mechanisms remain elusive [18,20,21]. In a previous study, we could show that Abi1 is strongly expressed in colorectal adenomas and carcinomas compared to healthy mucosa, and *KRAS-*mutated hyperplastic polyps show overexpression of the protein compared to their wild-type or *BRAF*-mutated counterparts [22]. Accordingly, overexpression of interaction partners of Abi1 (hnRNP K, N-WASP and WAVE2) has also been previously reported in colorectal carcinoma and CRC metastasis [23-26]. Given the role of Abi1 in actin dynamics, the goal of this study was to investigate its function in extracellular matrix degradation and invasion by CRC cells. Furthermore, we examined the effect of STI571 on Abi1 phosphorylation, metalloproteinase secretion, ECM degradation and colorectal cancer cell invasion.

## Results

### Abi1 mRNA expression patterns in gastrointestinal adenocarcinomas

**Figure 1:**
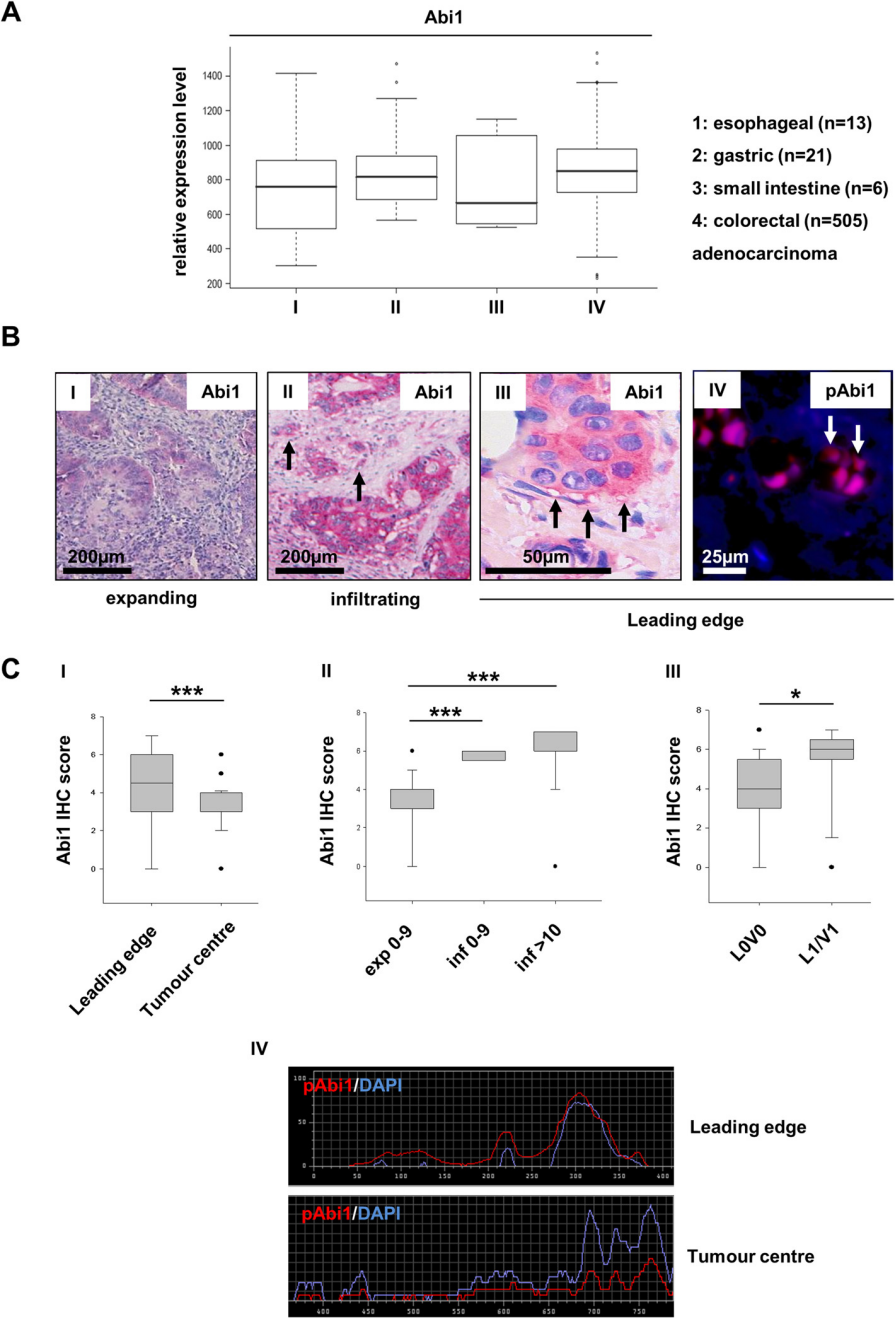
**Abi1 expression analyses. A**, *in silico* gene expression analysis using the GeneSapiens database [31] shows no significant differences in Abi1 gene expression among adenocarcinomas of gastrointestinal origin. **B**, representative microphotographs of Abi1 immunohistochemistry at the invasive margin of expanding (I) and infiltrating (II) CRC. Tumour cell budding in (II) is indicated by arrows. Greater magnification (III) shows strong Abi1 expression in cytoplasm and podia-like cellular protrusions (arrows). Immunofluorescence staining for phosphorylated Abi1 (p435) shows strong positivity in dissociating tumour cells at the invasive margin (IV). **C**, statistical evaluation of Abi1 IHC staining scores shows significant higher expression scores at the leading edge compared to tumour centre (I) as well as in tumours with infiltrating growth and high-grade tumour cell budding compared to tumours with expanding growth and low-grade budding (II). Moreover, Abi1 expression score is significantly associated with lymph and/or blood vessel infiltration by the tumour (III). Quantification of staining intensities (IV) shows stronger cytoplasmic as well as nuclear positivity for phosphorylated Abi1 (pY435) at the invasive margin compared to the tumour centre. IHC, immunohistochemistry; exp, expanding growth; inf, infiltrating growth; 0–9, 10: intensity of tumour cell budding in an x250 visual field. Scale bars as indicated. *p < 0.05; ***p < 0.001.

Quantitative Abi1 gene expression data for oesophageal (n = 13), gastric (n = 21), small intestinal (n = 6) and colorectal (n = 505) adenocarcinomas obtained from the GeneSapiens transcriptomics database showed comparable expression levels between tumour entities (Figure 1A). Median relative expression levels ranged from about 680 (small intestine adenocarcinoma) to 840 (colorectal carcinoma) arbitrary units.

### Clinic-pathologic sample characteristics

56 colorectal carcinoma surgical specimens were included in the study. All UICC stages, tumour localizations and histopathologic differentiations were represented among the sample cohort (Table 1). Lymph vessel infiltration was present in 19 cases (34%), while blood vessel infiltration was present in 10 tumours (18%). 36% of tumours showed an infiltrating growth pattern; 16 tumours (29%) displayed high-grade tumour cell budding at the leading edge. Statistical analysis (Fisher’s exact test) revealed a significant correlation between infiltrating growth pattern and high-grade tumour cell budding (p < 0.001) and confirmed the association between both infiltrating tumour growth and high-grade tumour cell budding and the presence of lymph or blood vessel invasion by the tumour (L1/V1, p = 0.019 and p = 0.011). 7 tumours (13%) showed loss of mismatch repair protein expression that was significantly associated with expanding, but not infiltrating growth pattern (p = 0.042). Activating *KRAS* or *BRAF* mutations were present in 42% and 4% of samples, respectively.

**Table 1:** Clinic-pathologic sample characteristics

**No. of patients**	**56**
**No. of samples**	**56 (100%)**
**Median age (years)**	72 ± 10.9
**Gender**	
**Male**	33 (59%)
**Female**	23 (41%)
**Location (n = 55)**	
**Right**	16 (29%)
**Left**	39 (71%)
**Grading**	
**Well**	6 (11%)
**Moderate**	24 (43%)
**Poor**	26 (46%)
**Growth pattern**	
**Expanding**	36 (64%)
**Infiltrating**	20 (36%)
**Budding intensity**	
**Low-grade**	40 (71%)
**High-grade**	16 (29%)
**Lymph vessel infiltration**	
**L0**	37 (66%)
**L1**	19 (34%)
**Blood vessel infiltration**	
**V0**	46 (82%)
**V1**	10 (18%)
**MMR protein expression**	
**MMR proteins expressed**	49 (87%)
**Loss of MLH1 + PMS2**	6 (11%)
**Loss of PMS2**	1 (2%)
***KRAS *** **status (n = 55)**	
***KRAS *** **wild-type**	32 (58%)
***KRAS *** **G12D**	9 (16%)
***KRAS *** **G12V**	7 (13%)
***KRAS *** **G12C**	1 (2%)
***KRAS *** **G12S**	1 (2%)
***KRAS *** **G13D**	5 (9%)
***BRAF *** **status**	
***BRAF *** **wild-type**	54 (96%)
***BRAF *** **V600E**	2 (4%)
**UICC Stage (n = 55)**	
**I**	16 (29%)
**II**	15 (27%)
**III**	17 (31%)
**IV**	7 (13%)

### Expression of Abi1 at the invasion front of colorectal cancer

Immunohistochemistry for Abi1 showed strong expression of the protein at the invasive margin of infiltrating, but not expanding CRC (Figure 1B I-III); statistical analysis revealed significant higher Abi1 staining score at the leading edge of the tumours compared to tumour centre (Figure 1C I; p < 0.001). There was significant overexpression of the protein in tumours displaying an infiltrating growth pattern and high-grade tumour cell budding compared to expanding tumours with a “pushing border” configuration (Figure 1B and 1C II, p < 0.001). Abi1 expression correlated with lymph or blood vessel invasion by the tumour (L1V1 status, Figure 1C III; p = 0.027). Immunofluorescence staining and quantification of staining intensities with an antibody against a phosphorylated isoform of Abi1 (pY435) showed strong nuclear and cytoplasmic positivity in dissociated tumor cells at the invasion front, but only weak staining signals in the tumour body (representative images, Figure 1B IV and Figure 1C IV).

### Expression and phosphorylation of Abi1 in CHD1 cells

**Figure 2:**
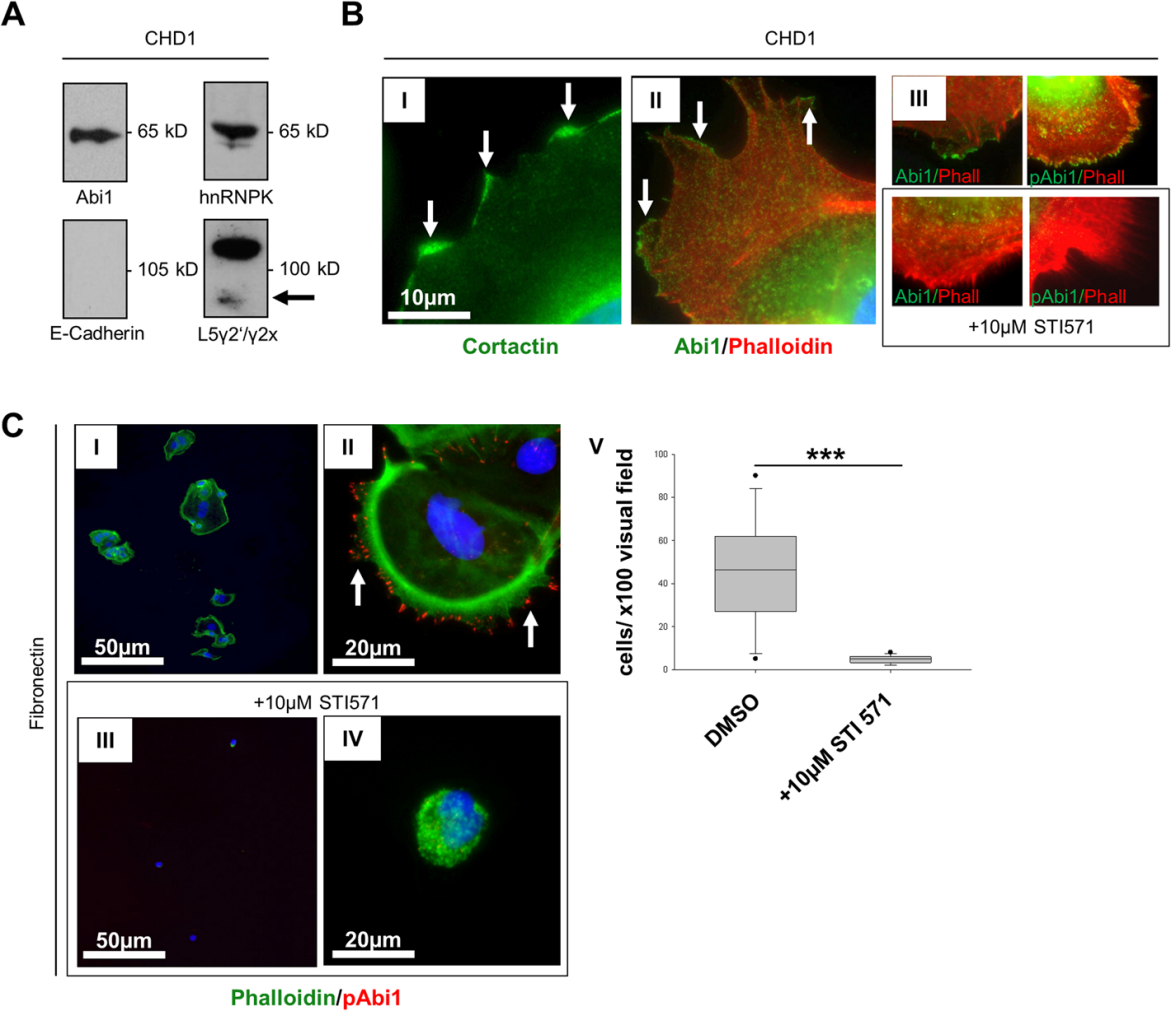
**Abi1 expression and subcellular localization in CHD1 colorectal carcinoma cells. A**, western immunoblotting of CHD1 whole cell lysate shows expression of Abi1 and hnRNP K as well as a 100/85 kD double-band for Laminin5, but no detectable levels of E-Cadherin. **B**, IF microscopy shows peripheral, rim-like distribution of Cortactin (I) and Abi1 (II). Signals for both Abi1 and Y435-phosphorylated Abi1 in the cell periphery, but not in the perinuclear region, are extinct after treatment with 10 μM STI571 (III). **C**, fibronectin adhesion assay shows cellular attachment with lamellipodial protrusions (I) and peripheral positivity for Y435-phosphorylated Abi1 (II) under control conditions, but only few attached cells (III) with condensed actin and negativity for pAbi1 after treatment with 10 μM STI571 (IV). Cell attachment was significantly reduced after treatment with STI571 (V). DMSO, dimethyl sulfoxide. Scale bars as indicated. ***p < 0.001.

CHD1 colorectal carcinoma cells are positive for Abi1, hnRNP K and Laminin5γ2 in Western immunoblotting (Figure 2A and Additional file 1: Figure S1A). The antibody against Laminin5 detected two bands migrating at 100kD (L5γ2′) and 85kD (L5γ2x), indicating cleavage of the protein [27]; E-cadherin was not expressed at a detectable level in CHD1 whole cell lysate. These findings could be confirmed in IF microscopy (Additional file 1: Figure S1B). Further immunofluorescence analyses showed localization of Cortactin and Abi1 to the outer rim of lamellipodia-like cellular protrusions (Figure 2B I and II). Immunofluorescence staining with an antibody against Y435-phosphorylated Abi1 showed strand-like positivity along the growth axis of cellular protrusions (Figure 2B III); treatment with 10 μM of the Abl tyrosine kinase inhibitor STI571 markedly reduced Abi1 and pAbi1 positivity in peripheral cellular compartments with remaining central (perinuclear) positivity for Abi1.

### Fibronectin cell adhesion assay

When seeded onto fibronectin-coated coverslips, CHD1 cells showed outgrowth of broad-based lamellipodia with peripheral, strand-like positivity for phosphorylated Abi1 (Figure 2C I and II). 10 μM STI571 significantly impaired lamellipodia formation and cellular adhesion on fibronectin (Figure 2C III-V, p < 0.001).

### Gelatine-based ECM degradation assay

**Figure 3:**
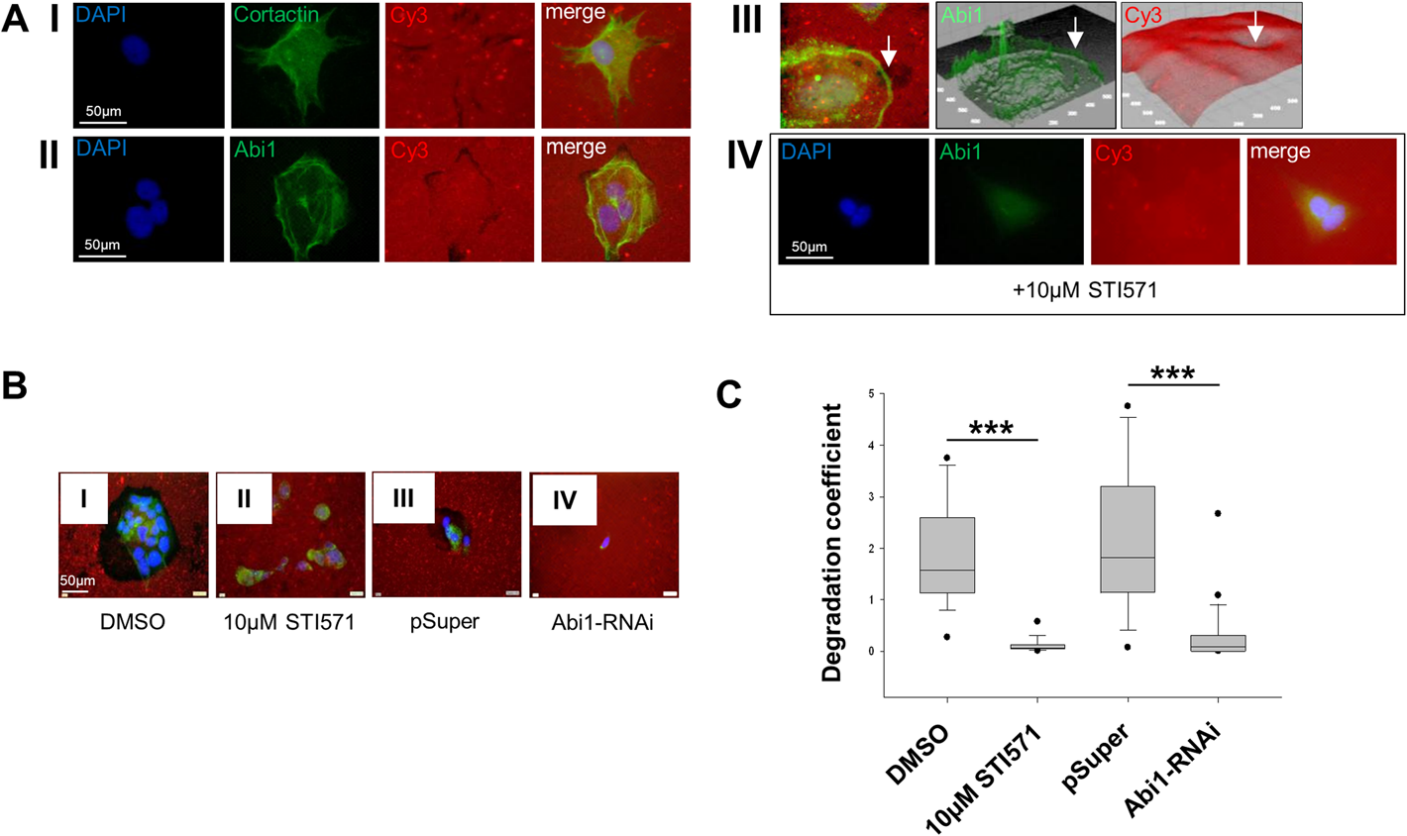
**Gelatine-based ECM degradation assay. A**, CHD1 cells seeded on Cy3-conjugated gelatine matrix show cytoplasmic and peripheral positivity for Cortactin (I) and Abi1 (II) at the sites of matrix degradation. This is confirmed in 3D surface reconstruction for Abi1 (III, arrow). Application of 10 μM STI 571 leads to loss of Abi1 staining in cell periphery together with lack of gelatine degradation (IV). **B and C**, Application of STI571 in a concentration of 10 μM (I: DMSO control, II: STI571) as well as Abi1 RNAi knockdown (III: pSuper vector control, IV: Abi1 RNAi) inhibits ECM degradation as shown by a significant reduction in degradation coefficient. DMSO, dimethyl sulfoxide. Scale bars as indicated. ***p < 0.001.

When CHD1-cells were seeded out on fluorescent gelatine-coated coverslips, Cortactin and Abi1 localized to peripheral foci of matrix degradation (Figure 3A I and II). This was confirmed in 3D surface reconstruction, where rim-like Abi1 positivity in the cell periphery co-localized with sites of matrix cleavage (Figure 3A III). Application of 10 μM STI571 led to a complete arrest in matrix degradation (Figure 3A IV).

### Effect of tyrosine kinase inhibition and Abi1 RNAi knockdown on ECM degradation

To further investigate the role of (phosphorylated) Abi1 in ECM degradation, CHD1 cells were seeded out on fluorescent gelatine-coated coverslips and either treated with 10 μM STI571/DMSO or transfected with RNAi targeting Abi1/vector control; after 24 h, degradation area was measured and degradation coefficients were calculated (see “Materials and methods” section for details). Statistical analysis revealed that 10 μM STI571 as well as Abi1 RNAi knockdown significantly reduced the ability of CHD1 cells to lyse extracellular matrix compared to DMSO-treated or vector control-transfected cells (Figure 3B and C; p < 0.001).

### Effect of tyrosine kinase inhibition on Abi1 phosphorylation, matrix metalloproteinase 9 secretion and tumour cell invasion

**Figure 4:**
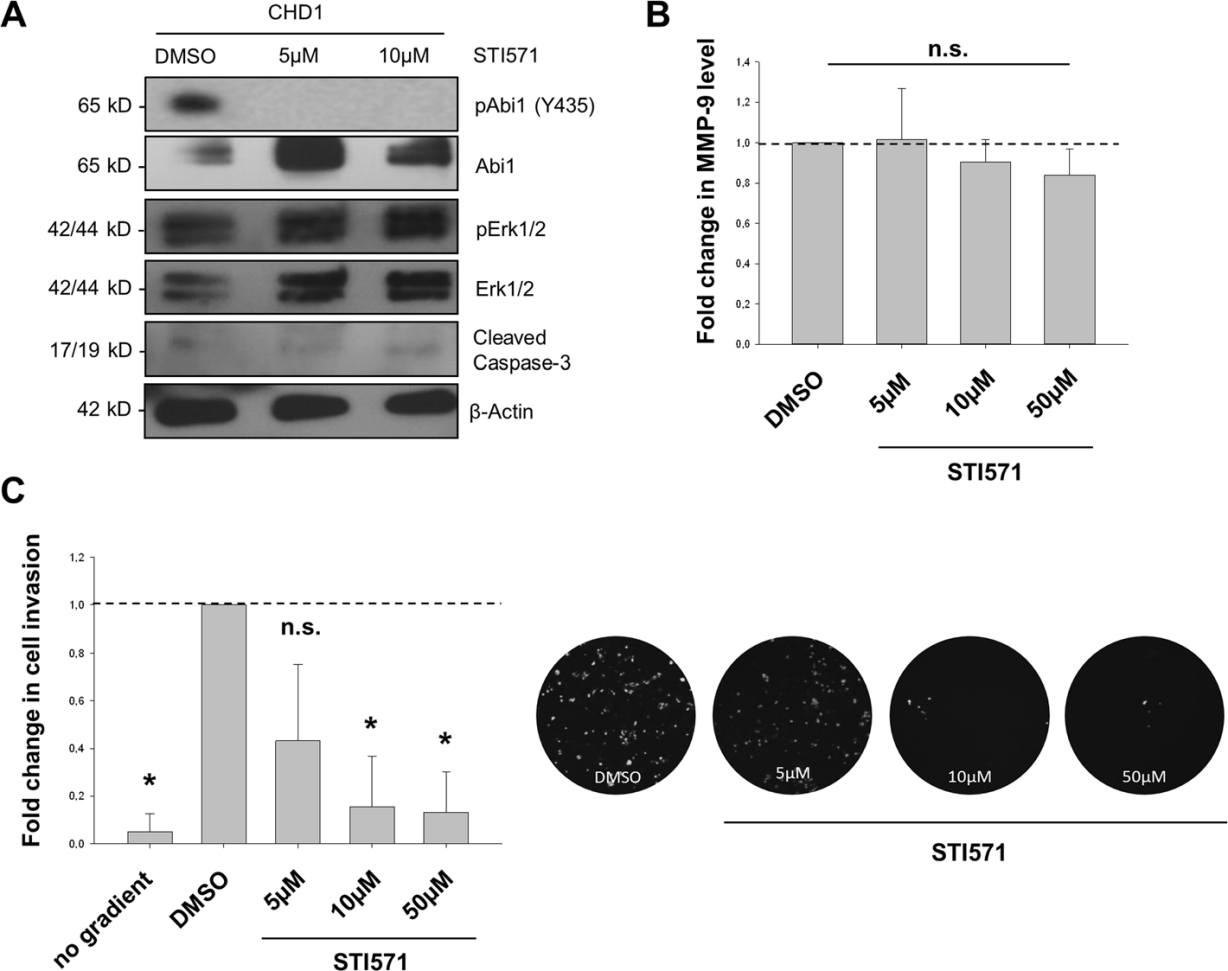
**Functional role of Abi1 phosphorylation in MMP-9 secretion and CRC cell invasion. A**, western immunoblotting of CHD1 cell lysate after control (DMSO) treatment or application of STI571 as indicated confirms inhibition of Abi1 Y435-phosphorylation through STI571, while levels of total Abi1, phospho-Erk1/2 and total Erk1/2 slightly increase upon STI treatment. Levels of cleaved caspase-3 are barely detectable. **B**, MMP-9 ELISA from cell culture supernatant after control (DMSO) treatment or application of STI571 shows a non-significant decrease in MMP-9 secretion after treatment with 5-50 μM STI571. **C**, increasing concentrations of STI571 inhibit CHD1 CRC cell invasion through reconstituted basement membrane in a dose-dependent manner. Representative microphotographs (right) show DAPI-counterstained cells that invaded through the sealed pores and were harvested from the lower chamber. DMSO, dimethyl sulfoxide; MMP-9, matrix metalloproteinase 9; n.s., not significant. *p < 0.05.

To confirm inhibition of Abi1 Y435-phosphorylation as well as specificity of STI571, we performed western immunoblotting after treatment with DMSO control or STI571. The band for phosphorylated Abi1 that was observed under control conditions was extinct after treatment with 5 and 10 μM STI571, while total Abi1 was strongly expressed (Figure 4A and Additional file 1: Figure S3A). There was no decrease, but a slight increase in Erk1/2 expression and phosphorylation, while protein levels of cleaved caspase-3 (CC-3) were barely detectable upon treatment with 5 and 10 μM STI571 (Figure 4A); application of 50 μM STI571 led to a strong increase in CC-3 expression (Additional file 1: Figure S3B). Beta-actin was used as a loading control.Changes in matrix metalloproteinase 9 (MMP-9) secretion were assessed using ELISA (Figure 4B) in cell culture supernatant from CHD1 cells that had been treated with STI571 or DMSO control as indicated. There was a slight decrease in MMP-9 secretion that was not statistically significant (p = 0.162).The ability of CHD1 cells to invade through a reconstituted basement membrane under increasing concentrations of STI571 was investigated applying the ECMatrix Cell Invasion Assay (EMD Millipore, Darmstadt, GER). Invaded cells were spun on a glass slide, counterstained with DAPI and counted automatically. 10 μM of STI571 reduced the invasive potential of CHD1 cells up to 80% (Figure 4C; p < 0.05).

## Discussion

Abelson interactor 1 (Abi1), a 65kD substrate of the eponymous Abelson tyrosine kinase (c-Abl), has been initially described as a regulator of actin dynamics and synaptic maturation in neurons [7,8,28]. In recent studies, overexpression of the protein has been linked to increased metastatic potential in breast and ovarian cancer, and several studies described an inhibitory effect of low concentrations of the c-Abl inhibitor STI571 on colorectal carcinoma cell invasion [18,20,21,29,30]. Our own group found overexpression of Abi1 in *KRAS*-mutated colonic precursor lesions and during colon carcinogenesis [22]; therefore, the aim of this study was to evaluate the expression and functional role of Abi1 in colorectal cancer (CRC) invasion as a prerequisite to tumour cell invasion and metastasis.

Gene expression levels were assessed using data from 545 tissue samples from the GeneSapiens *in silico* database [31] and showed no significant differences in Abi1 gene expression among adenocarcinomas of gastrointestinal origin. This finding is consistent with protein expression data obtained from the human protein atlas [32], another *in silico* database for tissue microarray-based protein expression patterns [33,34]. In that database, 86% of gastric and colorectal tumour specimens showed moderate to strong Abi1 staining intensity with the identical antibody that was used in the present study. Taken together, these large-scale expression analyses confirm the strong expression of Abi1 that we previously reported for CRC among diverse adenocarcinomas of the gastrointestinal tract [22]. However, Abi1 mRNA as well as protein expression data reveals great intra- and intertumoural heterogeneity. Therefore, we analysed Abi1 expression at the leading edge and in the tumour centre of 56 invasive CRCs and found that expression of the protein correlated significantly with infiltrating growth pattern and high-grade tumour cell budding, both characteristics being widely accepted to be associated with aggressive behaviour and poor prognosis in CRC [2,3]. We could confirm the correlation between infiltrative growth and high-grade tumour cell budding as well as lymph or blood vessel invasion by the tumour in our sample set, supporting the assumption that these morphologic features herald an aggressive tumour phenotype. Lymphatic and blood vessel invasion, representing significant prognostic variables in CRC, were independently associated with strong expression of Abi1 at the invasive margin of the tumours [35]. These findings are consistent with results obtained from other tumour entities, since it has been shown that overexpression of Abi1 is associated with early recurrence and worse survival in breast cancer; in ovarian cancer, Abi1 is an essential factor in a protein tri-complex indispensable for metastatic capability of tumour cells [29,30]. Moreover, immunofluorescence microscopy revealed a strong staining signal for a phosphorylated isoform of Abi1 (Y435) at the leading edge of infiltrating tumours with high expression of Abi1, indicating a role for Abi1 tyrosine phosphorylation in CRC cell invasion. To further investigate the functional role of Abi1 in CRC, we analysed expression and subcellular localization of the protein in CHD1 cells carrying an activating *KRAS* G13D mutation. Initially, the cell line had been selected because of its high Abi1 expression level [22], but in the present study, additional immunoblotting experiments showed cleavage of Laminin5γ2 and loss of E-cadherin expression in CHD1 cells. Both features are consistent with a pro-migratory, epithelial-mesenchymal-transition-like cellular phenotype that might be linked to constitutively active Ras signalling [36,37]. Accordingly, HDC9 *KRAS/BRAF* wild-type colorectal carcinoma cells - that weakly express Abi1 [22] - display high levels of E-cadherin and no cleavage of Laminin5 indicated by a single y2′ band migrating at 100–105 kD (Additional file 1: Figure S1A).

Immunofluorescence microscopy showed localization of Abi1 to a peripheral rim around lamellipodia-like cellular protrusions in cultured CHD1 cells, a distribution pattern comparable to the established invadopodia marker Cortactin [4]. The phosphorylated isoform of Abi1 (Abi1-pY435) was detected in strand-like alignments along broad-based cellular protrusions, and both peripheral staining signals were extinct after treatment with 10 μM of the Abl kinase inhibitor STI571 (Glivec®). Furthermore, this treatment prevented CHD1 cells from firmly attaching to fibronectin-covered surfaces. To verify the results from IF microscopy, we performed additional immunoblotting experiments and could confirm that the band for Y435-phosphorylated Abi1 was extinct after treatment with STI571, while levels of total Abi1 as well as total and phosphorylated Erk1/2 were even slightly elevated. These findings support the results from IF staining, where Abi1 remained strongly expressed upon STI571 treatment, and point towards a cellular redistribution of Abi1 rather than a decrease in protein expression upon kinase inhibition. Furthermore, as shown by Erk1/2 phosphorylation, signalling activity along the central MAPK pathway was not inhibited, but slightly enhanced by STI571 in the given concentrations. This is consistent with our previous results showing Abi1 upregulation upon Ras-MAPK signalling in colorectal precursor lesions, and might point towards an alternative signalling route via MAPK upon application of STI [22]. Western immunoblotting for cleaved caspase-3 gave no evidence for the induction of apoptosis upon application of 5 or 10 μM STI571, while there was strong immunoreactivity upon 50 μM of the drug, consistent with literature data [21]. It has to be recognised, however, while our results do show a decrease in Abi1 Y435-phosphorylation upon STI571 especially in the cell periphery that might be due to c-Abl inhibition, we did not prove that this tyrosine residue is in fact phosphorylated by c-Abl. This question should be addressed in further experiments applying knockout of Abl and/or site-directed mutagenesis.

**Figure 5:**
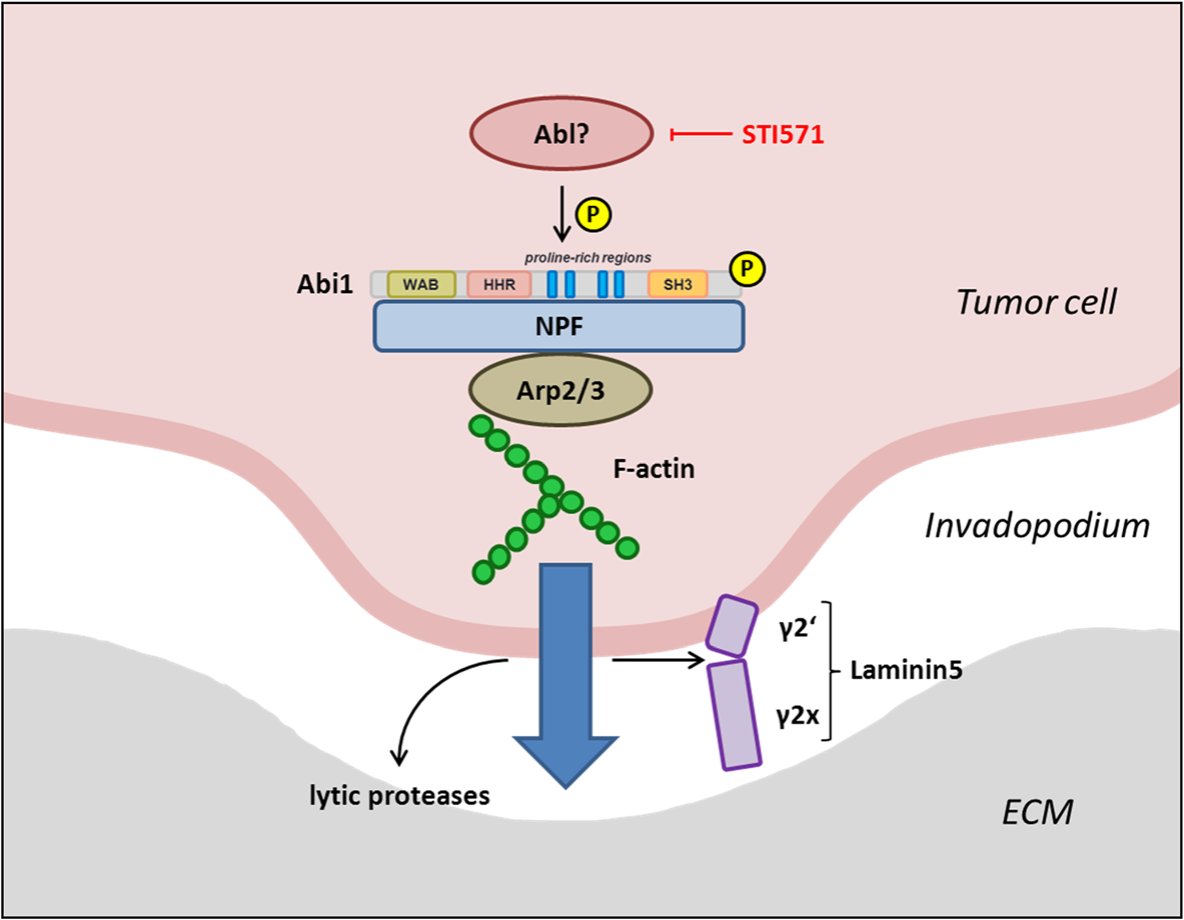
**A possible pathway for the functional role of Abi1 phosphorylation in colorectal carcinoma invasion.** Y435-Phosphorylation of Abi1 through Abl or another kinase that is targeted by STI571 enhances activity of class I and/or class II NPFs, thus facilitating actin branching and invadopodia formation. Secretion of lytic proteinases at the site of invadopodia formation leads to ECM degradation and cleavage of Laminin5, which supports local tumour cell invasion. *Abl, Abelson tyrosine kinase; Abi1, Abelson interactor 1; WAB, Wave-binding domain; HHR, homeobox homology region; SH3, src homology domain 3; NPF, nucleation-promoting factor; Arp2/3, actin-related protein complex; F-actin, filamentous actin; ECM, extracellular matrix.*

Invadopodia show a branched actin network closely related to the architecture observed in lamellipodia, but they are able to degrade ECM through secretion of lytic proteinases [4]. When CHD1 cells were seeded upon a fluorescent gelatine matrix, Abi1 and Cortactin localized to peripheral foci of matrix degradation, and application of STI571 as well as RNAi knockdown of Abi1 eliminated the ability of CHD1 cells to lyse the ECM as shown by a significant decrease in degradation coefficients. This is consistent with the described role of Abi1 in breast cancer cell invadopodia formation and might explain the inhibition of colorectal cancer cell migration and invasion through STI571 that has been previously shown [16,20]. Therefore, a model where Abi1 and Cortactin act cooperatively in invadopodia at the leading edge of colorectal carcinoma is quite conceivable; phosphorylated Abi1 would then support local actin reorganization via activation of nucleation-promoting factors, giving rise to a branched actin network required for ECM attachment and invadopodia formation (schematically depicted in Figure 5) [4,16]. This model is supported by the fact that upregulation of Abi1-interacting formins and class I NPFs has been reported to be associated with enhanced metastasis in CRC, and increased MMP9 secretion upon Abi1 overexpression has been shown in breast cancer [16,25,38]. However, in the present study, we could not show a significant decrease in MMP-9 secretion after STI571 treatment, why we conclude that the impact of Abi1 phosphorylation seems to lie in invadopodia formation rather than MMP-9 secretion by CRC cells. It might be discussed if other proteases play a central role during tumour cell invasion in CRC. One hint might be the observed cleavage of Laminin5, since this process is in most part mediated by MMP-1 and MMP-7, and only in little part by MMP-9, in colon cancer [36,39]. In our model, an increase in the local concentration of such lytic enzymes would lead to ECM dissolution and cleavage of Laminin5 (schematically depicted in Figure 5); accordingly, associations between high MMP-1 expression levels and both presence of liver metastases and poor prognosis have been reported in CRC [40,41].

Finally, we show that tumour cell invasion through a reconstituted basement membrane was diminished up to 80% after application of 10 μM STI571. These results are in line with findings from other authors, who described a remarkable decrease in cell migration capacity in response to STI treatment in colon cancer, and indicate that the antiinvasive function of STI571 might at least in part be mediated by inhibition of Abi1 phosphorylation that impairs invadopodia formation by CRC cells [18,20,21].

## Conclusions

Our results show for the first time that Abi1 is overexpressed at the invasive edge of colorectal carcinomas that display an aggressive phenotype; furthermore, phosphorylated Abi1 as well as Cortactin localize to foci of matrix degradation in the tumour cell periphery, while treatment with STI571 leads to a depletion of Y435-phosphorylated Abi1 in this compartment and suppresses tumour cell attachment to fibronectin as well as ECM degradation and tumour cell invasion in vitro. Finally, our findings support previously published data indicating that STI571 might be a pharmacological option to prevent the gain of an invasive phenotype in colorectal cancer.

## Materials and methods

### GeneSapiens database

To evaluate Abi1 gene expression patterns among different adenocarcinomas of the gastrointestinal tract, we obtained publicly available expression data for oesophageal (n = 13), gastric (n = 21), small intestine (n = 6) and colorectal (n = 505) adenocarcinomas from the GeneSapiens database [31,42]. This database contains data from 5 Affymetrix array generations (about 10 000 samples, average of 11 500 genes per sample) subdivided into healthy, malignant, and non-tumour disease tissue.

### Patient samples, clinical data and ethics statement

All patient samples have been submitted to the Institute of Pathology, Bundeswehrkrankenhaus Ulm, for routine diagnostic purposes between 2006 and 2012. Clinic-pathological data including TNM/UICC stages, tumour differentiation, infiltration patterns and presence of blood vessel invasion was obtained from the pathology report archive of the Institute of Pathology, Bundeswehrkrankenhaus Ulm, and are summarized in Table 1. All samples have been anonymised thoroughly for the use in the study. This study has been approved by the local ethics committee of the University of Ulm (No. 162/13).

### KRAS/BRAF mutation testing and MMR protein immunohistochemistry

Mutation status of *KRAS* (codons 12, 13 and 61) and *BRAF* (codon 600) was performed using *KRAS/BRAF* strip assays (Vienna Labs, Vienna, AUT and AID diagnostics, Strassberg, GER) as previously described [22]. Shortly, DNA was extracted from 30 mm-thick slides that were cut off paraffin blocks, xylene treated and ethanol washed. PCR amplification of *KRAS* and *BRAF* with biotinylated primers and hybridization-based mutation analysis was done according to the manufacturers’ protocols. Immunohistochemistry for mismatch repair (MMR) proteins MLH-1, MSH-2, MLH-6 and PMS-2 was performed on BenchMark Autostainer (Ventana, Tucson, USA) using prediluted mouse monoclonal antibodies (Ventana, Tucson, USA).

### Evaluation of invasion patterns and immunohistochemical analysis

Invasion pattern of each tumour was assessed applying the Jass classification modified by Ueno et al., since this scheme has been shown to represent a good index to estimate the aggressiveness of colorectal adenocarcinoma [2,3]. The classification scheme divides invasive margins into “expanding” (pushing/well circumscribed) or “infiltrating” (diffuse, widespread penetration) and takes into account the grade of tumour “budding”. As in the original publication, budding was defined as isolated cells or clusters of less than five cancer cells; the number of budding foci was counted in a field where budding intensity was considered maximal using a ×25 objective lens. 0–9 foci were defined as low-grade, while ≥10 foci were defined as high-grade tumour budding.

Abi1 immunohistochemistry was performed on 1–2 slides that displayed the invasive margin of the tumours using a mouse monoclonal antibody (clone 1B9, MBL, Woburn, USA; conc. 1:200). Staining intensity was evaluated at the invasion front of the tumours using the scoring system described by Carpenter et al. [23]. The intensity of immunostaining was graded as negative = 0, weak = 1, moderate = 2 or strong = 3. The proportion of positively stained cells was assessed as no cells = 0, 1–25% of cells = 1, 26–50% = 2, 51–75% = 3 and 76–100% = 4. The numbers representing intensity and percentage of stained cells were added together and the result will be referred to as score.

### Cell culture, pharmacological treatment, MMP-9 ELISA and immunoblotting

CHD1 cells were obtained from the Institute of Pathology, University of Ulm, and have been previously published [22,43]. Cells were kept under sterile conditions in RPMI-1640 medium supplemented with 10% FCS. STI571 was dissolved in DMSO and applied in the indicated concentrations 24 or 48 h prior to cell lysis/fixation. Cell culture supernatants were collected and delivered to matrix-metalloproteinase 9 ELISA using the Human MMP-9 Quantikine ELISA Kit (R&D Systems, Wiesbaden, Germany) while adherent cells were lysed in standard lysis buffer containing protease and phosphatase inhibitors (Cell Signaling Tech., Boston, USA). Immunoblotting was performed according to standard methods using the following antibodies: mouse monoclonal to Abi1 (1:200), MBL, Woburn, USA; mouse monoclonal to hnRNP K (1:500), Sigma-Aldrich, St. Louis, USA; rabbit polyclonal to E-Cadherin (1:250), Sigma-Aldrich, St. Louis, USA; rabbit polyclonal to Laminin5γ2 (1:250), Sigma-Aldrich, St. Louis, USA; rabbit monoclonal to Abi1-pY435 (1:250), Abcam, Cambridge, UK; rabbit monoclonal to p44/42 (Erk1/2, 1:1000), phospho-p44/42 (phospho-Erk1/2, 1:2000) and cleaved caspase-3 (1:1000), all from Cell Signaling Tech., Boston, USA; mouse monoclonal to actin (1:1000), Thermo Scientific, Rockford, USA.

### Immunofluorescence (IF) staining and fibronectin adhesion assay

For immunofluorescence (IF) staining’s, CHD1 colorectal carcinoma cells were seeded onto 4-well chamber slides (Nunc, Wiesbaden, GER), and staining was carried out as previously described [44]. In short, cells were fixed in ice-cold 4% paraformaldehyde, permeabilised with 0.2% Triton-X 100 and incubated with 5% FCS in PBS. Alternatively, FFPE slides from tumour centre and invasive margin were deparaffinised, permeabilised and blocked with 5% FCS in PBS. Afterwards, slides were incubated with one or two of the following primary antibodies for 1 h at room temperature: mouse monoclonal to Abi1 (1:200, MBL, Woburn, USA); rabbit monoclonal to Cortactin (1:250, Sigma-Aldrich, St. Louis, USA); rabbit polyclonal to Abi1-pY435 (1:250, Abcam, Cambridge, UK); mouse monoclonal to hnRNP K (1:250), Sigma-Aldrich, St. Louis, USA; rabbit polyclonal to E-Cadherin (1:250), Sigma-Aldrich, St. Louis, USA; rabbit polyclonal to Laminin5γ2 (1:250), Sigma-Aldrich, St. Louis, USA. After washing, slides were incubated with secondary antibodies (goat polyclonal anti-mouse, Texas Red- conjugated, Abcam, Cambridge, UK; goat polyclonal anti-rabbit, DyLight®488- conjugated, Vector Laboratories, Burlingame, USA; goat polyclonal anti-mouse IgM Antibody, Cy2-conjugated, Biozol, Eching, GER; goat polyclonal anti-mouse IgG (H + L), F(ab’)2 Fragment, Alexa Fluor® 488-conjugate, Life technologies, Darmstadt, GER). For phalloidin staining, FITC/ TexasRed-coupled Phalloidin (Life technologies, Darmstadt, GER) was added after 30 min incubation at RT in the dark. Slides were mounted in Vectashield aqueous mounting medium containing DAPI (Vector Laboratories, Burlingame, USA). For the fibronectin adhesion assay, chamber slides were covered with Human Plasma Fibronectin (Life technologies, Darmstadt, GER, conc. 25 μg/ml) for 2 h at 37°C. Cells were seeded in serum-free medium with either 10 μM STI571 or DMSO (control). 6 hours after seeding, slides were 6 times rinsed with PBS, and attached cells underwent fixation and immunofluorescence staining as described above. Image acquisition as well as quantification of IF staining intensities was performed using a Zeiss Axioplan microscope and Isis software (MetaSystems, Altlussheim, GER); for fibronectin adhesion assays, quantification of attached cells in 12 visual fields (×10 objective lens) was done using Image J image analysis software (National Institutes of Health, Bethesda, USA).

### ECM degradation assay

For visualization of ECM degradation, the QCM™ Gelatine Invadopodia Assay (EMD Millipore, Darmstadt, GER) was applied according to the manufacturer’s protocol. Briefly, 8-well-chamberslides (Nunc, Wiesbaden, GER) were covered with Poly-L-Lysine, Glutaraldehyde and Cy3-labeled fluorescent gelatine. CHD1 colorectal carcinoma cells were seeded in fresh growth medium at 500 μL/well and allowed to attach for 6 h before RNAi transfection/pharmacological treatment. After another 36 h (transfection)/24 h (treatment), medium was removed and slides were fixed in 4% PFA for immunofluorescence staining as described above. Qualitative and quantitative image analysis was done using Image J image analysis software (National Institutes of Health, Bethesda, USA) [45]. 3D surface plots were generated using the 3D surface plot add-in for Image J (Kai Uwe Bartel, HTW Berlin, GER). Quantification of extracellular matrix degradation was done using ImageJ analysis software as previously described [46]. High threshold was set for the DAPI channel and counted as “particles” for total cell count and the Cy2/Alexa488/FITC signal for total cell surface area per field of view. Conversely, low intensity threshold was set for diminished Cy3-gelatin signal to enable quantification of total degradation area. The ratio between degradation area and cell area was defined as the degradation coefficient (DC). For quantification of matrix degradation after RNAi knockdown and pSuper vector control, only the degradation coefficient of cells that showed cytoplasmic pSuper marker fluorescence was calculated. For DMSO- or STI571-treated cells, Phalloidin counterstaining was used to visualize cell surface area.

### Small interference RNA (RNAi) experiments

Knockdown of Abi1 was achieved by RNAi according to published methods using the pSuper vector (OligoEngine, Seattle, USA). Complementary oligonucleotides were annealed and inserted into the Hind III/Bgl II sites of the pSUPER vector. Initially, the Abi1 construct had been designed complementary to *rattus norvegicus* Abi1 mRNA [GenBank NM_024397]. NCBI blast sequence analysis revealed 100% homology with *homo sapiens* Abi1 over a span of 16 nucleotides (sequence in *italics*), a feature which has been previously reported to be an even more effective trigger of RNA induced silencing complex (RISC) formation than 19-nt-long cores [47]; Abi1 RNAi: 5′-GAT CCC C*AG GCT ACA GAC AAG AG*G AAT TCA AGA GAT TCC TCT TGT CTG TAG CCT TTT TTG GAA A-3′ (sense) and 5′- AGC TTT TCC AAA AAA GGC TAC AGA CAA GAG GAA TCT CTT GAA TTC CTC TTG TCT GTA GCC TGG G-3′ (antisense). Protein knockdown efficacy of the construct in human CHD1 cells was tested using immunofluorescence analysis and western immunoblotting (Additional file 1: Figure S2A and B). Transfections were performed using the Optifect transfection reagent (Qiagen, Hilden, GER) according to the manufacturer’s protocol in serum-free medium (4 μl per μg DNA).

### Cell invasion assay

To evaluate ability of colorectal carcinoma cells to invade into ECM, cells were seeded onto the polycarbonate membranes of cell culture inserts with 8 μm-pores sealed with ECMatrix, a reconstituted basement membrane matrix derived from Engelbreth Holm-Swarm (EHS) mouse tumour (ECMatrix Cell Invasion Assay, EMD Millipore, Darmstadt, GER) [48]. Cells were then treated with STI571 in the indicated concentrations for 48 hrs; for each treatment, n = 6 experiments were performed. Invasive tumour cells that have invaded through the ECM-covered pores along an FCS gradient to the bottom of the polycarbonate membrane and into the outer chamber and were trypsinised, spun on glass slides (Menzel, Braunschweig, GER) and counterstained with DAPI. Quantification of cells in 6 visual fields (×10 objective lens) was done using Image J image analysis software (National Institutes of Health, Bethesda, USA).

### Statistics

Differences in Abi1 IHC scores between invasion front/ tumour centre, tumours with different invasion patterns (expanding/low-grade budding, expanding/high-grade budding, infiltrating/low-grade budding and infiltrating/high-grade budding) and tumours with and without lymphovascular infiltration were analysed using the Kruskal-Wallis test followed by Dunn’s multiple comparison post-test (SigmaPlot 12.0, Systat Software, Erkrath, Germany). For quantification of ECM degradation, mean degradation coefficients for 10–15 visual fields for each treatment/transfection were calculated. The differences between degradation coefficients after DMSO control/STI571 treatments and vector control/RNAi knockdown as well as between numbers of attached/invading cells in fibronectin adhesion/ECMatrix cell invasion assay were tested for significance performing nonparametric ANOVA (Kruskal-Wallis) test using SigmaPlot 12.0 (Systat Software, Erkrath, Germany) with Dunn’s multiple comparison post-test, where applicable. *P* values <0.05 were regarded as statistically significant.

### Consent

All cases that are reported in the manuscript were collected for histologic examination and diagnosis purpose and thoroughly anonymized for the use in this study. Informed consent was therefore not needed to be obtained. This study was approved by the instutional ethics committee of the University of Ulm (decision no. 162/13) and conforms to the current guidelines of the German Ethics Council.

## Abbreviations

Abi1: Abelson interactor 1; Abl: Abelson tyrosine kinase; Arp2/3: Actin-related protein 2/3; *BRAF*: V-Raf murine sarcoma viral oncogene homolog B1; CC-3: Cleaved caspase-3; CRC: Colorectal carcinoma; DMSO: Dimethyl sulfoxide; ECM: Extracellular matrix; F-actin: Filamentous actin; FCS: Fetal calf serum; HHR: Homeobox homology region; hnRNP K: Heterogeneous nuclear ribonucleoprotein K; HSPC300 (syn. BRICK1): SCAR/WAVE actin-nucleating complex subunit; *KRAS*: Kirsten rat sarcoma; MAPK: Mitogen-associated protein kinase; MMP: Matrix metalloproteinase; Nap: NCK-associated protein 1; NPF: Nucleation-promoting factor; PI3K: Phosphatidylinositol-3-kinase; SH3: Src homology domain 3; Sra (syn. PIR121): p53 inducible protein; UICC: *Union international contre le cancer*; WAB: Wave-binding domain; (N-) WASP/WAVE: (neural) Wiskott-Aldrich syndrome protein/ verprolin homologous.

## Competing interests

The authors declare that they have no competing interests.

## Authors’ contributions

KS and KK carried out (immuno-) histologic analyses. KS and SB carried out *in vitro* analyses (cell culture, matrix degradation and cell invasion assays). KS carried out immunofluorescence microscopy. KS, JS and CP performed western immunoblotting. KS, JL and PM participated in the design of the study, and KS performed the statistical analyses. KS, JL, JS, VM and PM conceived of the study, and participated in its design and coordination and helped to draft the manuscript. All authors read and approved the final manuscript.

## Supplementary Material

Additional file 1: Figure S1 Further analysis of Laminin5, E-cadherin and hnRNP K expression in CHD1 and HDC9 cells. **A**, western immunoblottig against Laminin5 (left), blots are shown after short (15 s) and long exposure (10 min) and E-Cadherin (right). **B**, immunofluorescence stainings of hnRNP K/Phalloidin, Laminin5/Phalloidin and E-cadherin/Phalloidin. *Scale bars as indicated.***Figure S2.** Verification of Abi1 RNAi knockdown in CHD1 CRC colorectal carcinoma cells. **A**, by immunofluorescence microscopy. **B**, by western immunoblotting (compared to pSuper vector control). *Scale bars as indicated.***Figure S3.** Western immunoblotting of CHD1 cells after application of DMSO control as well as 5, 10 **(A)** and 50 μM STI571 **(B)**. **A**, with antibodies against Abi1 and β-Actin (loading control). **B**, with an antibody against cleaved caspase-3 (CC-3). While CC-3 is barely detectable after DMSO or after application of 5 or 10 μM STI, there is strong immunoreactivity upon 50 μM STI571. *DMSO, dimethyl sulfoxide.* Click here for file
