# Supplementary figures and images for: Expression and Y435-phosphorylation of Abelson interactor 1 (Abi1) promotes tumour cell adhesion, extracellular matrix degradation and invasion by colorectal carcinoma cells
Source: Mol Cancer. 2014 Jun 9;13:145. doi: 10.1186/1476-4598-13-145 (PMC4066275; doi:10.1186/1476-4598-13-145)

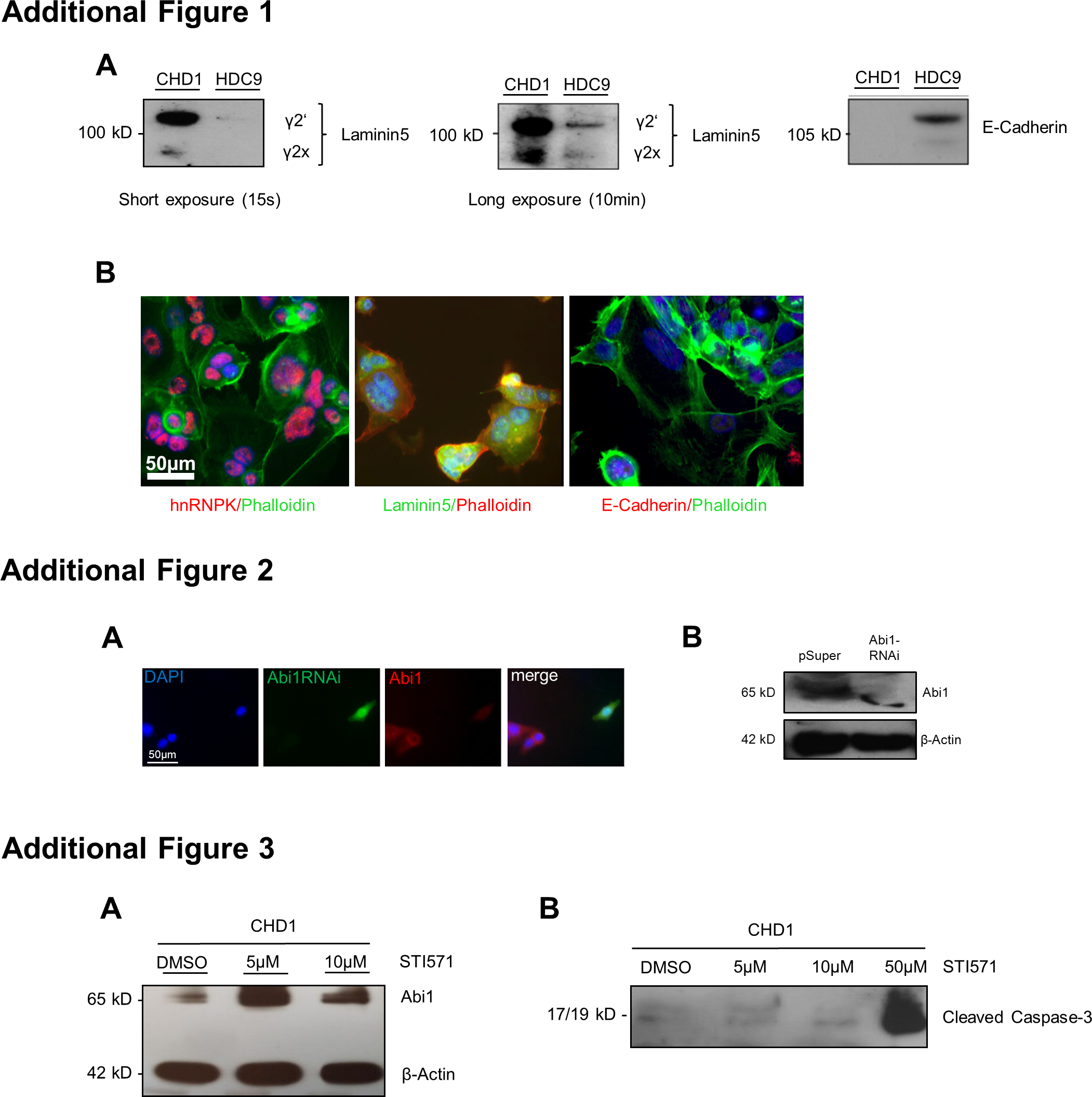

Supplement: Additional file 1: Figure S1 — Further analysis of Laminin5, E-cadherin and hnRNP K expression in CHD1 and HDC9 cells. A, western immunoblottig against Laminin5 (left), blots are shown after short (15 s) and long exposure (10 min) and E-Cadherin (right). B, immunofluorescence stainings of hnRNP K/Phalloidin, Laminin5/Phalloidin and E-cadherin/Phalloidin. Scale bars as indicated.Figure S2. Verification of Abi1 RNAi knockdown in CHD1 CRC colorectal carcinoma cells. A, by immunofluorescence microscopy. B, by western immunoblotting (compared to pSuper vector control). Scale bars as indicated.Figure S3. Western immunoblotting of CHD1 cells after application of DMSO control as well as 5, 10 (A) and 50 μM STI571 (B). A, with antibodies against Abi1 and β-Actin (loading control). B, with an antibody against cleaved caspase-3 (CC-3). While CC-3 is barely detectable after DMSO or after application of 5 or 10 μM STI, there is strong immunoreactivity upon 50 μM STI571. DMSO, dimethyl sulfoxide. [file 1476-4598-13-145-S1.tiff]
